# Case Report: Immunotherapy for low-grade myofibroblastic sarcoma of the pharynx

**DOI:** 10.3389/fimmu.2023.1190210

**Published:** 2023-07-04

**Authors:** Bao Sun, Zhiying Luo, Ping Liu, Yan He, Shasha He, Wenhui Liu

**Affiliations:** ^1^ Department of Pharmacy, The Second Xiangya Hospital, Central South University, Changsha, China; ^2^ Institute of Clinical Pharmacy, Central South University, Changsha, China; ^3^ Department of Oncology, The Second Xiangya Hospital, Central South University, Changsha, China

**Keywords:** low-grade myofibroblastic sarcoma, pharynx, recurrence and multiple metastases, pembrolizumab, immunotherapy

## Abstract

Low-grade myofibroblastic sarcoma (LGMS) characterized by the increased proliferation of myofibroblasts is a rare type of malignant myofibroblastic tumor that frequently occurs in the head and neck region. Presently, there is no consensus regarding the treatment of LGMS. Here, we report a rare case of LGMS of the pharynx in a 40-year-old male admitted to our hospital. The patient underwent resection for a right metastatic lesion and parapharyngeal mass. However, he had recurrence and multiple metastases without a surgical indication. Then the patient received the treatment of anlotinib plus pembrolizumab for 4 cycles, and there was a partial response (PR) to the treatment. Due to the adverse reaction of anlotinib, the patient subsequently received monotherapy of pembrolizumab for 22 cycles and achieved a complete response (CR). As the first case report of the immunotherapy for LGMS, our study highlights that this strategy may be of great significance to the treatment of LGMS.

## 1 Introduction

Low-grade myofibroblastic sarcoma (LGMS) defined as a distinct atypical myofibroblastic tumor is a rare solid infiltrative soft tissue tumor, often with predilection for the head and neck region (1). LGMS is mainly located in deep soft tissues, and it has a high recurrence rate but low metastatic potential (2). In addition, LGMS most commonly occurs in adults, mainly in men with an average age of 40 (3, 4). The diagnosis of LGMS is usually based on the histological and immunohistochemical findings (5). Due to the rarity of LGMS, however, the standardization of its treatment remains unclear. Generally, surgery is the primary treatment for LGMS.

Herein, we present a case of LGMS occurring in the pharynx. After the resection of the metastatic lesion, the patient had recurrence and multiple metastases without a surgical indication. Thus, he received the combined treatment of pembrolizumab and anlotinib, and subsequent monotherapy of pembrolizumab. To the best of our knowledge, this is the first case report of LGMS with a good response to immunotherapy.

## 2 Case presentation

**Figure 1:**
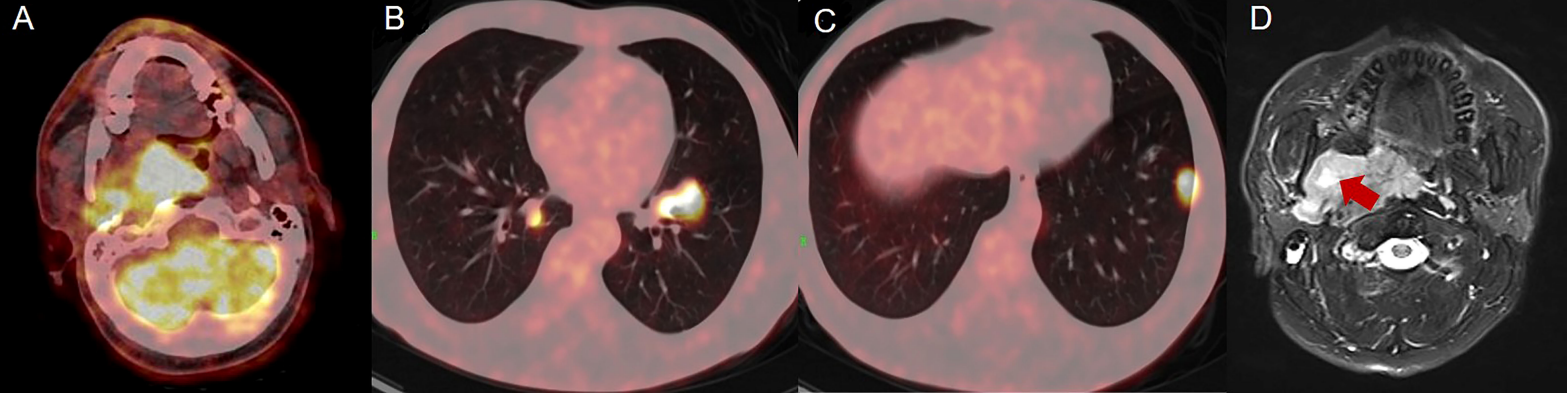
Positron emission tomography and computed tomography (PET/CT) and magnetic resonance images (MRI) of lesion and mass. **(A–C)** PET/CT revealed a mass in right lateral wall of the pharynx, along with lung metastases. **(D)** MRI showed a soft tissue mass with a size of 7.5 cm × 7.5 cm × 3 cm.

**Figure 2:**
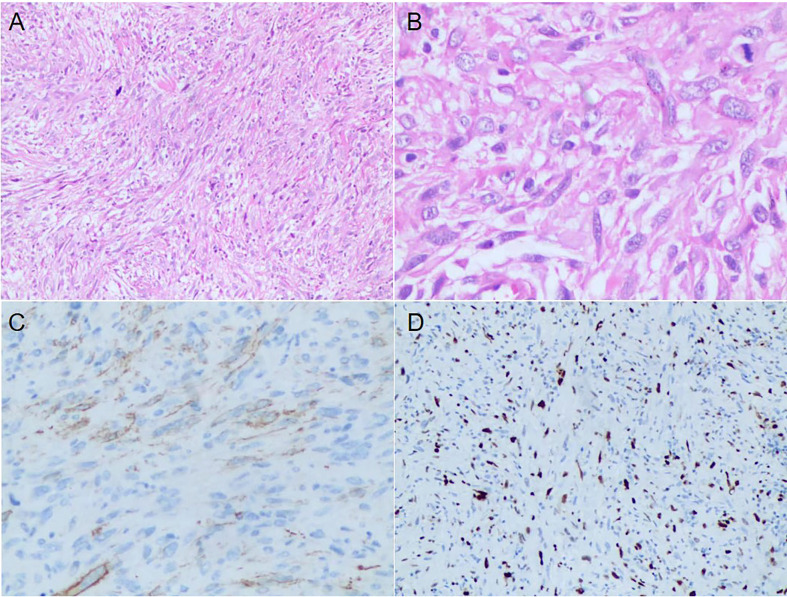
Histopathological and Immunohistochemical results of the excisional biopsy. **(A)** The tumor cells were fusiform, arranged in fascicles or storiform growth patterns (HE, ×100). **(B)** A few mitoses and atypical cells with irregular nuclei were observed (HE, ×400). **(C, D)** Immunohistochemical results showed positive staining for α-SMA and Ki-67 with a 40% proliferation index.

**Figure 3:**
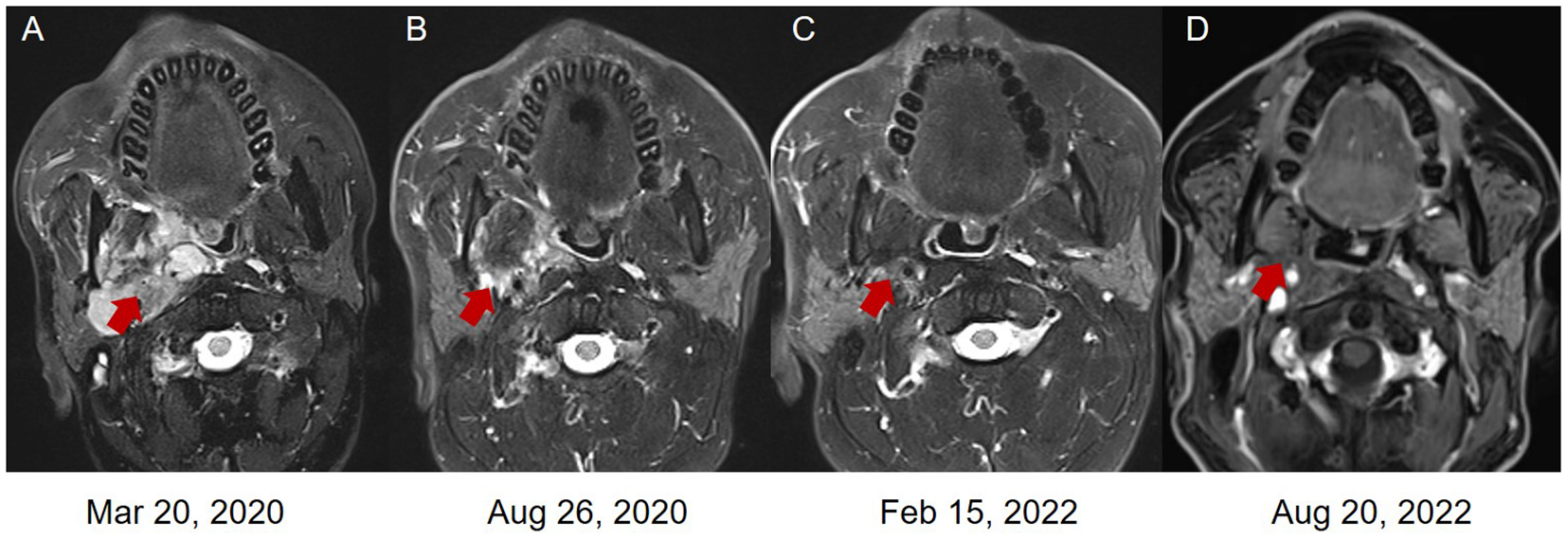
The magnetic resonance images (MRI) of the LGMS. **(A)** In March 2020, new multiple lymphatic metastases were observed. **(B)** In August 2020, the patient obtained a PR after the combined treatment of pembrolizumab and anlotinib. The patient obtained a PR **(C)** and CR **(D)** after the monotherapy of pembrolizumab.

**Figure 4:**
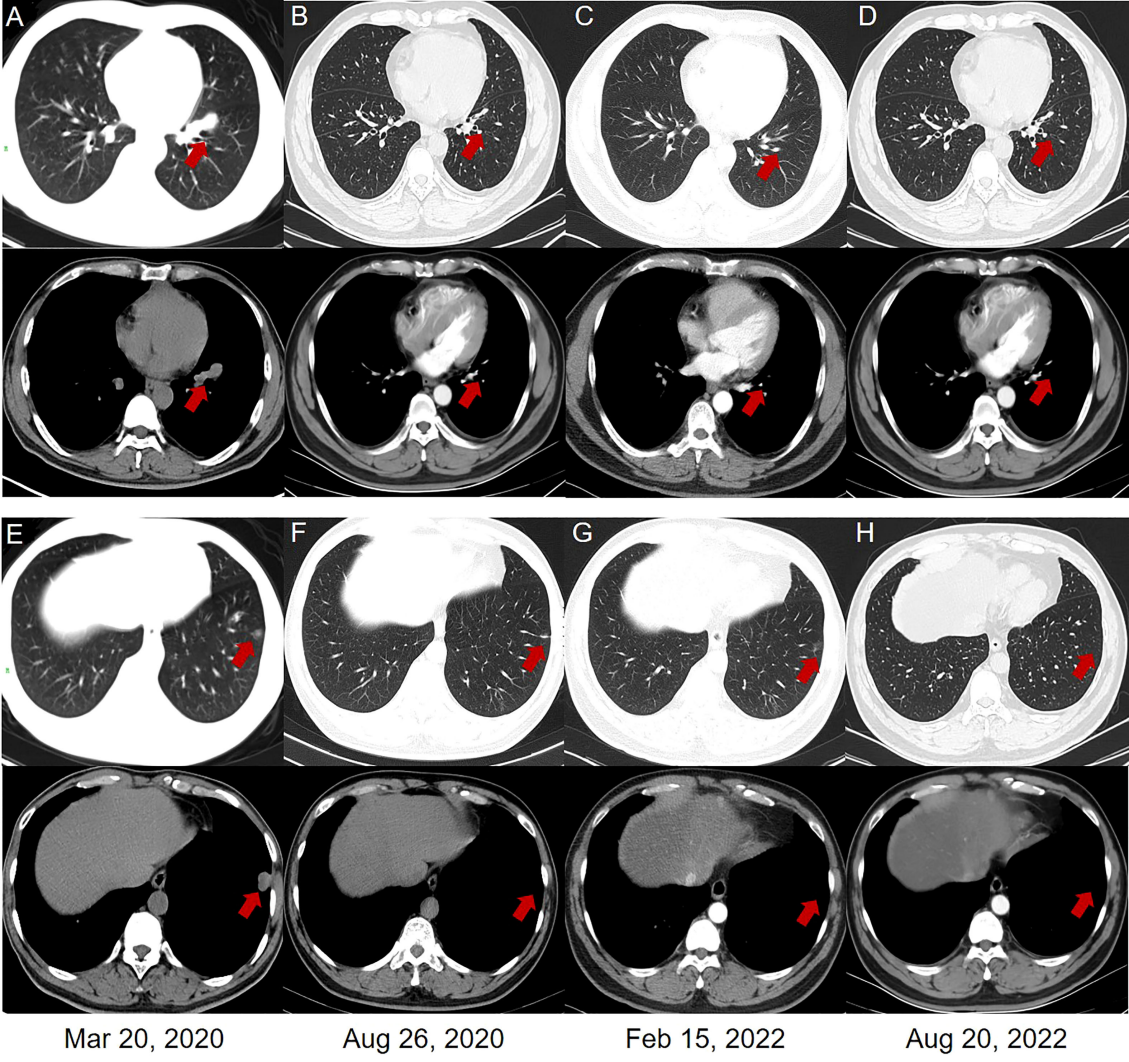
Chest computed tomography (CT) scans. **(A, E)** In March 2020, multiple metastases of lung and mediastinal window were observed. **(B, F)** In August 2020, a PR was revealed in the lung and mediastinal window of the patient after the combined treatment of pembrolizumab and anlotinib. The patient obtained a PR **(C, G)** and CR **(D, H)** in the lung and mediastinal window after the monotherapy of pembrolizumab.

In January 2020, a 40-year-old male was admitted to our hospital with obvious right pharyngeal foreign body sensation and pharyngeal discomfort. The patient had no history of other disease and denied the family history. Positron emission tomography and computed tomography (PET/CT) showed a mass in the right lateral wall of the pharynx, along with lung metastases (**Figures 1A–C**). Magnetic resonance images (MRI) revealed a soft tissue mass in the right parapharyngeal space (7.5 cm × 7.5 cm × 3 cm) (**Figure 1D**). Furthermore, there were multiple lymphadenopathies in bilateral neck region. The patient underwent a surgical resection of metastatic lesion and parapharyngeal mass of the right pharynx on January 15, 2020. Histological examination showed spindle cells with minimally atypia arranged with fascicular or storiform growth patterns in the fibrous stroma, suggestive of low-grade spindle cell sarcoma (**Figures 2A, B**). Regarding immunohistochemical staining, there is positive result for smooth muscle actin (SMA), but a negative result for caldesmon and desmin, with a Ki-67 index of approximately 40% (**Figures 2C, D**). The diagnosis of LGMS was established based on the histological features together with immunohistochemical findings. In March 2020, multiple metastases were observed again in the patient’s lymph nodes and lungs (**Figures 3A**, **4A, E**). According to the advice of multi-disciplinary treatment (MDT), the patient did not have a surgical indication.

Starting from April 2020, the patient received 4 cycles of combined therapy with pembrolizumab (200 mg, q21d) and anlotinib (12 mg, qd, d1-14, q3w) and obtained a PR to drug treatment. Considering the hypertensive side effect induced by anlotinib (systolic blood pressure: 180 mmHg; diastolic blood pressure: 110 mmHg), the patient stopped using anlotinib in the follow-up treatment. Subsequently, 22 cycles of pembrolizumab monotherapy (200 mg, q21d) were performed as maintenance therapy from August 2020 to August 2022. Based on the new response evaluation criteria in solid tumors (RECIST) guideline (version 1.1) (6), the patient obtained a PR in August 2020 and February 2022, respectively (50% and 67% decrease in the sum of diameters of target lesions, respectively) (**Figures 3B, C**, **4B, C, F, G**). Not until the last follow-up in August 2022, the patients obtained a CR in August 2022 (disappearance of all target lesions) (**Figures 3D**, **4D, H**). The timeline of treatment administration from the episode of care was presented in **Supplementary Figure 1**.

## 3 Discussion

In this report, we presented a successful case of a patient with LGMS who obtained a good response to immunotherapy. The patient was initially diagnosed with LGMS, along with lung metastases. Of note, he had another recurrence and multiple metastases after surgery without a surgical indication. Emerging evidence demonstrated that targeted therapy, immunotherapy or a combination of both were important approaches in treating soft-tissue sarcoma (STS) (7–9). In the present case, we observed a durable effect in the patient treated with immunotherapy, highlighting the importance of the immunotherapy in the treatment of LGMS.

As a rare STS, LGMS is a low-grade malignant tumor derived from mesenchymal myofibroblasts, characterized by its local recurrence and occasional metastasis (1). A previous report revealed that LGMS was mainly located in the head and neck region, especially in the oral cavity, and generalized that local recurrence of LGMS was 26.7%, and distant metastasis was 4.4% (10). As yet, the diagnostic features of LGMS remained challenging. The differential diagnosis for this tumor included nodular fasciitis, low-grade fibrosarcoma, inflammatory myofibroblastic tumor, well-differentiated osteosarcoma, desmoplastic fibroma, leiomyosarcoma, and fibromatosis. The histopathologic resemblance of LGMS to fibromatosis and myofibroma was often a source of diagnostic confusion. In terms of the LGMS in the upper aerodigestive tract, Meng et al. (11) reported that misdiagnosis might occur in small and superficial biopsy samples due to the diverse histologic appearance in the same tumor of myofibroblastic sarcoma. Given that a misinterpretation could result from the specimen being sampled from the tumor surface, Montebugnoli et al. (12) considered that an open incisional biopsy with subsequent histopathological evaluation must be performed. In addition to histologic similarities, LGMS might also be mistaken for nodular fasciitis because of its overlapping immunophenotypes (13). Immunohistochemical results showed that cases with myofibroblastic sarcomas were diffusely positive for at least one myogenic marker and vimentin, including muscle−specific actin and α−SMA (5). Several reports indicated that LGMS might be immunopositive for muscle−specific actin, α−SMA, calponin, fibronectin, and desmin (14, 15). Collectively, although histopathological analysis together with immunohistochemical results were usually considered to confirm the diagnosis of LGMS, the complete clinical features of LGMS were still unclear and needed further investigation.

The primary treatment for LGMS is surgical excision (5, 16). However, several studies suggested surgical excision combined with adjuvant therapy to prevent local recurrence (17, 18). Notably, a recent case series disclosed the association of local recurrence and the tissue invasion of LGMS with the surgical method. Surgical excision with wider safety margins was considered to minimize the risk of recurrence (17). However, wider safety margins were usually more problematic in the oropharynx than in other parts of the body. Besides, there is a controversy in postoperative therapy including radiotherapy and chemotherapy to prevent local recurrence. There was no recurrence for LGMS in the pancreas for five years after surgery and adjuvant chemotherapy (19), while other reports recommended that laryngeal and sacral LGMS were not sensitive to postoperative radiotherapy and chemotherapy (20, 21). Therefore, the selection of postoperative therapy might depend on the invasive region of LGMS and whether the tumor was completely resected.

The National Comprehensive Cancer Network (NCCN) guidelines supported that anthracycline-based chemotherapy was the standard first-line treatment for patients with STS (22). For individuals who failed first-line treatment, antiangiogenesis therapy was the promising strategy in the second-line treatment of advanced or metastatic STS. Anlotinib is a multitarget tyrosine kinase inhibitor (TKI) against tumor angiogenesis and tumor cell proliferation by targeting VEGFR, FGFR, PDGFR, and c-Kit simultaneously. A multi-centered phase IIB trial supported that anlotinib significantly prolonged median progression-free survival (PFS) from 1.47 to 6.26 months in patients with STS as a second-line or subsequent-line treatment (23). Another phase IIB trial conducted in the Chinese population also confirmed the positive efficacy of anlotinib in patients with advanced STS in a real-world setting (24). Furthermore, a recent clinical trial demonstrated that the combination of anlotinib and epirubicin followed by anlotinib treatment maintenance could serve as the first-line treatment for patients with advanced STS (25). On the other hand, immunotherapy was another possible strategy in the second-line treatment against advanced or metastatic STS. A phase II trial demonstrated that nivolumab combined with ipilimumab showed encouraging objective response rates, PFS and overall survival in certain sarcoma subtypes (8). Importantly, targeted therapy combined with immunotherapy has a synergistic effect on the disease (26). A recent report also showed that the combination of anlotinib and toripalimab was an effective therapy in advanced STS (27). In the present case, the patient could not tolerate anthracycline-based chemotherapy, thus we applied anlotinib combined with pembrolizumab, and the patient obtained a PR. However, the main serious adverse effects of anlotinib were hypertension and hand-foot skin reaction (24, 28, 29). Since the patient experienced hypertension (systolic blood pressure: 180 mmHg; diastolic blood pressure: 110 mmHg) after the combined treatment for 4 cycles, he stopped using anlotinib and subsequently switched to the monotherapy of pembrolizumab for 22 cycles. Ultimately, the patient reached CR.

The exact reason why monotherapy of pembrolizumab was effective in LGMS remained elusive. Pollack et al. identified the detailed overview of the immune microenvironment in sarcoma subtypes and found that high expression levels of genes related to antigen presentation and T‐cell infiltration, and T‐cell infiltration was significantly correlated with PD‐1 and PD‐L1 expression levels (30). Therefore, immunotherapy may exert effect through regulating gene expression related to antigen presentation and improving T-cell infiltration. Future studies are warranted to explore underlying cellular and molecular mechanisms of immunotherapy in LGMS.

To our knowledge, this is the first report of the immunotherapy for LGMS. Nevertheless, there exist several limitations in our report. Our report only provides preliminary results but does not figure out the specific reason for the effectiveness of immunotherapy. In addition, the elaborated mechanisms of immunotherapy need to be further clarified in the future.

## 4 Conclusion

In conclusion, our study sheds light that immunotherapy may be of great significance to the treatment of LGMS.

## Data availability statement

The original contributions presented in the study are included in the article/**Supplementary Material**. Further inquiries can be directed to the corresponding authors.

## Ethics statement

Written informed consent was obtained from the individual(s), and minor(s)’ legal guardian/next of kin, for the publication of any potentially identifiable images or data included in this article. Written informed consent was obtained from the participant/patient(s) for the publication of this case report.

## Author contributions

BS drafted the manuscript. ZL, PL and YH collected materials and prepared figures. WL and SH critically revised the final manuscript. All authors contributed to the article and approved the submitted version.

## Supplementary material

Supplementary Figure 1 The timeline of treatment administration from the episode of care.
