# Supplementary figures and images for: Case Report: Immunotherapy for low-grade myofibroblastic sarcoma of the pharynx
Source: Front Immunol. 2023 Jul 4;14:1190210. doi: 10.3389/fimmu.2023.1190210 (PMC10352614; doi:10.3389/fimmu.2023.1190210)

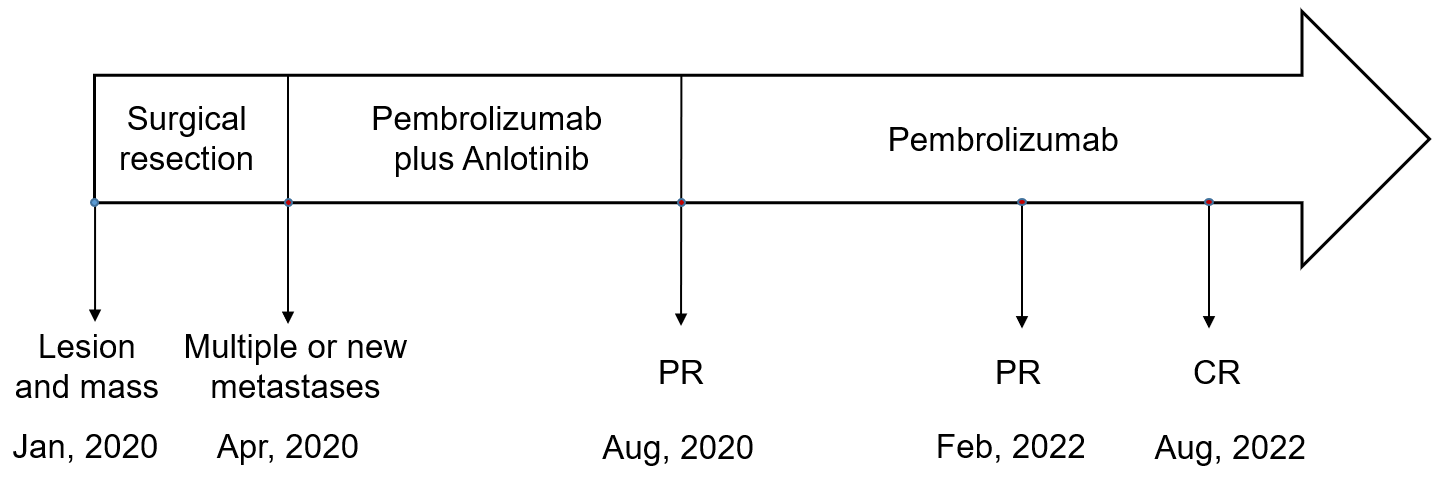

Supplement: Supplementary Figure 1 — The timeline of treatment administration from the episode of care. [file Image_1.tif]
